# Visualization and probability-based scoring of structural variants within repetitive sequences

**DOI:** 10.1093/bioinformatics/btu054

**Published:** 2014-02-04

**Authors:** Eitan Halper-Stromberg, Jared Steranka, Kathleen H. Burns, Sarven Sabunciyan, Rafael A. Irizarry

**Affiliations:** ^1^Department of Biostatistics, Bloomberg School of Public Health, Johns Hopkins University, ^2^Program in Human Genetics and Molecular Biology, Johns Hopkins University School of Medicine, ^3^Computational Bioscience Program, University of Colorado, Denver, ^4^Department of Molecular Biology and Genetics, ^5^Department of Oncology, ^6^The Sidney Kimmel Comprehensive Cancer Center, Johns Hopkins Hospital, ^7^Department of Pathology, Johns Hopkins University, ^8^High Throughput Biology Center, Johns Hopkins University School of Medicine, Johns Hopkins University, ^9^Center for Epigenetics, Johns Hopkins University School of Medicine, ^10^Department of Pediatrics, Johns Hopkins University School of Medicine, Baltimore, MD and ^11^Department of Biostatistics and Computational Biology, Dana Farber Cancer Institute, Boston, Massachusetts, MA, USA

## Abstract

**Motivation:** Repetitive sequences account for approximately half of the human genome. Accurately ascertaining sequences in these regions with next generation sequencers is challenging, and requires a different set of analytical techniques than for reads originating from unique sequences. Complicating the matter are repetitive regions subject to programmed rearrangements, as is the case with the antigen-binding domains in the Immunoglobulin (Ig) and T-cell receptor (TCR) loci.

**Results:** We developed a probability-based score and visualization method to aid in distinguishing true structural variants from alignment artifacts. We demonstrate the usefulness of this method in its ability to separate real structural variants from false positives generated with existing upstream analysis tools. We validated our approach using both target-capture and whole-genome experiments. Capture sequencing reads were generated from primary lymphoid tumors, cancer cell lines and an EBV-transformed lymphoblast cell line over the Ig and TCR loci. Whole-genome sequencing reads were from a lymphoblastoid cell-line.

**Availability:** We implement our method as an R package available at https://github.com/Eitan177/targetSeqView. Code to reproduce the figures and results are also available.

**Contact:** ehalper2@jhmi.edu

**Supplementary information:** Supplementary data are available at *Bioinformatics* online.

## 1 INTRODUCTION

Structural variants (SVs), including deletions, insertions, inversions and translocations, are known to contribute to a wide range of human phenotypes (Schinzel, 1988). High-throughput technology has facilitated exciting findings, associating SVs with multi-genic diseases like autism (Pinto, *et al.*, 2010) and schizophrenia (Stefansson *et al.*, 2008), and revealing genomic rearrangements in cancer (Molenaar *et al.*, 2012). Thousands of genomic loci in humans are now known to vary structurally. Next generation sequencing (NGS) is central to the field, evidenced by the high visibility of consortia that use NGS, like the 1000 Genomes Project (The 1000 Genomes Project Consortium, 2010).

Despite group efforts and recent advances, discovering and annotating the full landscape of SVs in humans is incomplete. This is in part owed to the inaccuracy of NGS in defining repetitive DNA. Repetitive DNA, stretches of nucleotides present in more than one copy in the haploid genome, accounts for about half of the human genome. These stretches may be sub-classified by length, copy number, base composition and linear organization, all of which are difficult to assay with NGS (Treangen and Salzberg, 2012).

Paired-end mapping, the most widely used NGS method for detecting SVs, has some ability to define SVs in repetitive sequences. The idea is to find read–pairs that map with an unexpected distance or orientation relative to one another, therefore implying an SV. When one or both ends of a read–pair map to the reference genome in multiple locations they provide evidence for multiple, contradictory structural arrangements**.** This is particularly problematic with NGS because a standard approach, among the most widely used aligners, is to randomly pick and report only one location for reads that map to multiple locations. This can easily lead to paired-end alignments that appear to be SVs when in fact the reads were from a contiguous piece of the reference genome. In this article we demonstrate how this is a substantial problem in practice and present a method that greatly helps ameliorate the problem.

Currently, a handful of tools, including HYDRA (Quinlan *et al.*, 2010), GASVPro (Sindi *et al.*, 2012) and VariationHunter (Hormozdiari *et al.*, 2010), attempt to resolve the inconsistencies generated from ambiguously mapped reads. HYDRA and VariationHunter choose one alignment per read from a set of possibilities to designate as correct. GASVPro weighs multiple mappings per read in proportion to their probability.

Despite clever algorithms, the accuracy of these tools is limited when the ends map ambiguously (this point will be demonstrated in Section 3.2). As a result, one must either discard results in repetitive DNA, or perform independent validation. One popular form of the latter involves visualizing alignments over candidates. Several viewers are available but there is a lack of options tailored to discriminating false SVs from true SVs (Koboldt *et al.*, 2012).

**Fig. 1.**
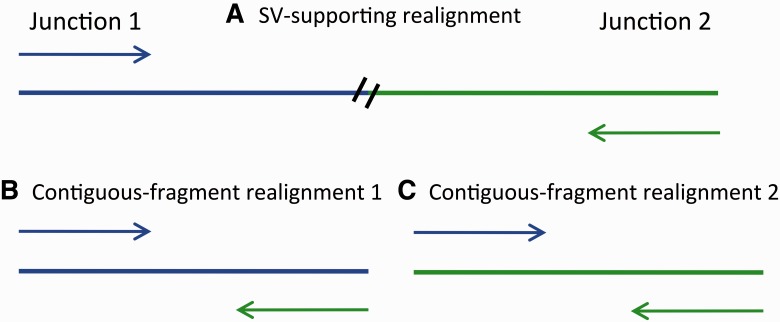
Schematic of three alignments considered (A) read–pair aligned so as to support the existence of an SV. Each read in the pair aligns to one side of the junction. Different colors indicate different loci within the genome (B) The same read–pair from (A) aligned so as to support a contiguous sequence fragment, generated by sequence from junction 1. (C) The same read–pair from (A) aligned so as to support a contiguous sequence fragment, generated by sequence from junction 2. The colors indicate that the read–pair supports the SV more so than either contiguous sequence fragment since in each of (B) and (C), one of the reads does not match the reference

We developed a probability-based score that can be used to prioritize candidate SVs. Our score weighs two contradictory hypotheses: read–pairs either belong to both the junctions of an SV, one read per junction or they belong to just one of the junctions (Fig. 1). Because univariate scores are sometimes too simplistic we also developed a visualization tool for alignments for candidate SVs. To avoid reinventing the wheel, the starting point of our method is the outputs of the existing SV discovery tools.

We illustrate the usefulness of our method on two experiments both of which provided a way to validate findings (described in detail in Section 2.2 and in the supplement). The first was a typical whole-genome sequencing experiment and the second was a target-capture sequencing on a region known to have a high prevelance of SVs (Halper-Stromberg *et al.*, 2013). For the whole-genome experiment we sequenced a diploid lymphobastoid cell-line from an anonymized female individual (Halper-Stromberg *et al.*, 2011). For the capture experiment we targeted sequences in the Ig and TCR loci in primary lymphoid cancer samples, cancer cell lines and an EBV transformed cell line. These loci, the building blocks of antigen binding domains in immunoglobulins and T-cell receptors, are characterized by consecutive homologous sequences. This repetitive, segmentally duplicated architecture makes these loci notoriously difficult to assay using NGS (Watson and Breden, 2012). These loci are an area of much interest because of their fundamental role in immunology and cancer (Mills *et al.*, 2003).

At the heart of our contribution to SV detection, our procedure filtered out false positives resulting from repetitive sequence in these data. For validation of our target-capture results we used PCR. Our target sequence was enriched for segmental duplications about six times above the genome-wide level. They represented 37% of the target sequence while they are present in 5.7% of the human genome. Of validated false positives in the target-capture sequences, 75% (29/38) contained breakpoints overlapping a segmental duplication. Importantly, we also identified true SVs within segmental duplications in our target-capture data. Of validated true positives in the target-capture, 50% (13/26) contained breakpoints that overlapped a segmental duplication. Segmental duplications were also a significant source of spurious results in our whole-genome data. Of validated false positives in the whole-genome data, 48% (73/151) contained breakpoints overlapping a segmental duplication while for true positives in the whole-genome data only 15% (6/39) did so. To validate these results we used read–depth-based CNV calls from three whole-genome sequencing runs of our lymphoblast sample. Our results demonstrate that our method effectively distinguishes artifacts from true SVs in repetitive loci.

## 2 METHODS

We realign reads to three reference sequences, one supporting the SV and two supporting contiguous fragments, one contiguous fragment for each side of the putative junction. We summarize whether alignments supporting the SV are better than both of the alignments supporting contiguous fragments with a probability-based score. We also visualize the three alignments to evaluate all of its aspects. In this section we present the main ideas leaving some details in Supplementary Material.

### 2.1 Generating a candidate SV list

For our target-capture experiment, we generated a list of candidate SVs using HYDRA, GASV (Sindi *et al.*, 2009) (the Supplementary Material explains why we did not use GASVPro with our target-capture data), and VariationHunter. For our whole-genome experiment we used these three tools as well as GASVPro. In both cases we mapped reads to the hg19 reference and preprocessed alignment files as required by each tool (details in the Supplementary Material).

For our target-capture experiment, we created a master list of follow-up candidates using HYDRA. This was the first software we used and we undertook validation immediately upon generating candidates. We generated candidates from GASV and VariationHunter primarily to compare their prioritization against our method, not to make unbiased comparisons (favors HYDRA) between the three existing methods.

For our whole-genome experiment, we created a master list of candidate SVs to interrogate for follow-up using results from all four tools, HYDRA, GASV, GASVPro and VariationHunter.

### 2.2 Validation data

To obtain our validation set for the target-capture experiment we substantially filtered the results of HYDRA in an attempt to retain only high confidence calls. Our filtering criteria required that a candidate: (i) be supported by at least five read–pairs, (ii) overlap with at least one of our bait sequences and (iii) be supported by read–pairs aligning with relatively few mismatches (supporting read–pairs were required to have a mean edit distance <2.6). Only 70 candidate junctions met these three criteria. We further processed this list down to 52 junctions, primarily with manual inspection to remove likely artifacts (described in further detail in the Supplementary Material). These 52 junctions were PCR validated. An additional 12 junctions representing canonical V(D)J recombination were not PCR validated but were assigned as true positives based on the fact that canonical V(D)J recombination is an expected result in our samples. Of the final list of 64 junctions, validation revealed that 26 (41%) were positives and 38 were negatives (59%).

To obtain validation for the whole-genome experiment read–depth-based deletion calls were generated using independent software on the results of three whole-genome sequencing runs on the same lymphoblast sample. We created two low coverage (6X) single-end datasets and one higher coverage (15X) paired-end dataset. Specifically, we generated read–depth-based deletion calls on all three of these samples using ERDs (Zhu *et al.*, 2012) and CNVnator (Abyzov *et al.*, 2011) on the paired-end sample and CNVnator on the single-end samples (ERDs does not work on low coverage samples). A candidate deletion was assigned as positive if it was identified by read–depth in the paired-end sample and at least one of the single-end samples (further details in the Supplementary Material).

We used these data to compare and contrast different approaches for SV detection in terms of sensitivity and specificity.

### 2.3 Realignment

We realigned all reads within candidate SV sites using Smith–Waterman-based alignment utilities provided through the Biostrings package (Pages *et al.*, 2013) in Bioconductor (Gentleman *et al.*, 2004), with parameters reflecting the probabilities of observing alignment errors, explained further in Section 2.4 and Supplementary Material ‘Smith–Waterman alignment parameters’ (Malde, 2008). Note that available short-read aligners are optimized for speed and memory efficiency as opposed to specifically returning the three alignments per read–pair most useful to determining the accuracy of an SV. Note also that SV callers depend on third-party alignment, e.g. Bowtie (Langmead *et al.*, 2009), Bowtie2 (Langmead and Salzberg, 2012), BWA (Li and Durbin, 2009), Novoalign (Novocraft, 2010) and MrFast (Alkan *et al.*, 2009), and are bound to the alignments in these third-party-generated files.

We recommend aligners that report multiple alignments per read. If multiple alignments are not returned, false positives will be reduced but at the cost of reduced sensitivity. Our method removes false positives but cannot recover false negatives. Random placement is well suited to read–depth analysis but it denies direct comparisons of the three alignment patterns we seek.

Understanding precisely how a particular aligner will behave with respect to repetitive sequences is outside the scope for most users and contributes to the popularity of ignoring repetitive loci (Yu *et al.*, 2012). The complexity in choosing aligner settings and the potential for missing alignments makes the case against dependence on primary alignments. We believe Smith–Waterman realignment of reads mapping to candidate SVs is a means to overcome the deficits of placing full faith in an alignment file.

### 2.4 Probability-based score

After the SV software generates long lists of candidates (most of them false positives) we need to isolate those that are likely to be SVs. The naïve approach of looking at quality metrics for the reported alignments does not work because some reported SVs might have been just as likely to come from a contiguous region, but this possibility was never considered. We developed a score-based relative evidence statistic: how much more likely is this candidate to be an SV as opposed to an uninteresting contiguous sequence. We will form a log-likelihood after defining a probability model.

We made several simplifying assumptions for our likelihood model similar to those made by SHRiMP (Rumble *et al.*, 2009). These include the assumption that mismatches and indels are independent of one another and each other, within and across reads. We also assume mismatch and indel rates are independent of genomic context. Further, we assume that read–pairs aligning at the junctions of a candidate SV either belong at both junctions, thereby supporting the SV, or they belong at only one of those junctions, thereby refuting the SV. We do not consider the scenario whereby read–pairs aligning at the junctions of one SV actually belong to a different SV (it is for this reason that we recommend an aligner that will report multiple alignments per read). With these assumptions in place we use a binomial distribution of mismatches and indels within our experiment, again similar to SHRiMP (Rumble *et al.*, 2009). Our model, unlike SHRiMP, accounts for varying error rates along reads. Position-specific error rates are known to affect Illumina short reads (Bravo and Irizarry, 2010). The ends of reads are most negatively affected due to the long cycle time of the first cycle and the tendency of phase errors to accumulate at the end of a run (Kircher *et al.*, 2009). To accommodate this phenomenon we use a different binomial distribution for each position within the reads.

For simplicity, our statistical model integrates only indels and mismatches because Illumina sequencing technology, in contrast to those reporting reads in color-space, does not allow mismatches to be easily categorized as SNPs or errors when evaluating single reads. Of note, we explored the possibility of separating mismatches into two separate categories, SNPs and sequencing errors, by using concordantly called mismatches at a given reference position across multiple reads (Supplementary Figure S1), but we do not distinguish SNPs from sequencing errors in the method presented here. Under our model the probability of a sequencer generating *m_p_* mismatches in *l* reads at read position *p* is:

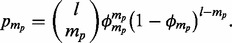

Where *l* is the number of reads, *m_p_* is the observed number of mismatches in all *l* reads at read–position *p* and 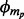 is the position-specific mismatch rate. Similarly, the probability of a sequencer generating *i_p_* indels in *l* reads at read–position *p* is:

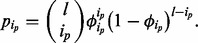

Where *l* is the number of reads, *i_p_* is the observed number of indels at read–position *p*, and 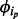 is the position-specific indel rate. We simplify calculations by assuming independence, as SHRiMP did: the probability of seeing *m_p_* and *i_p_* at a given read–position was their product. The probability of a sequencer generating a group of aligned reads, each with some observed number of mismatches and indels at each position, and assuming positions are independent of one another is then:

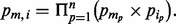



To estimate the rates of indels and mismatches within our experiments we sampled 100 000 concordant read–pairs, realigned these reads and assigned the mismatch and indel rates for each read–position to be the means of observed mismatches and indels in the realignments at each read–position, respectively. This estimation was performed separately for each experiment calculation of experiment-specific rates is a built-in feature of our tool.

Now to actually construct the score in practice, for each candidate SV, we first extract read–pairs from an alignment file that have one side mapping to each of the two loci indicated to be involved in the event. Second, we realign these reads to three reference sequences, one supporting the SV and two supporting contiguous fragments. Third, we compute the probability of each of these three alignments based upon a binomial model.

The score for the candidate SV is then the log likelihood comparing the probability of the rearranged reference sequence generating the observed reads versus the probability that the reads were generated from a contiguous section of the reference with respect to either side of the candidate junction.

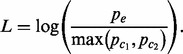



Here *p_e_* is the probability of a rearranged reference sequence generating a group of observed reads, 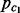 is the probability of a contiguous sequence taken from the 5′-side of candidate junction generating those reads, and 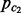 is the probability of a contiguous sequence taken from the 3′-side of a candidate junction generating those reads. Since we generate probabilities for each of the two possible contiguous fragments, we use only the probabilities from the fragment with the better alignments to construct the likelihood. This fragment is the more likely of the two possible contiguous sequences to be the true fragment generating the reads.

### 2.5 Visualization method

The three alignment configurations for each candidate SV are made into a picture to provide an intuitive representation of the data used to construct the likelihood. Realigned read–pairs are represented as gray bars, one read–pair per row, with black caps added to the 3′-end of each aligned read to signify the alignment orientation. Red dashes within the reads represent mismatches between the read and reference. Light blue within reads represent deletions, or a split-read. If the blue within a read crosses a junction, i.e. between the left and right pictures representing the 5′- and 3′-junctions of the SV, respectively, the read is a split-read. Deleted bases at the ends of reads are not shown, and appear as shortened bars. The three alignments are always shown with the alignment supporting the SV on top and the two alignments supporting contiguous sequences below.

## 3 RESULTS

We used our method with four SV finders, HYDRA, GASV, GASVPro and VariationHunter, to analyze sequences from whole-genome and target–capture data. The final validation set from the whole-genome data included 190 distinct deletions, 39 (21%) positives and 151 (79%) negatives and 64 distinct SVs, from the target-capture, 26 (41%) positives and 39 (59%) negatives.

Our sequencing library for the target–capture experiment included fragments selectively captured from V(D)J loci on chromosomes 2, 7, 14 and 22 in a panel of neoplastic B and T lymphocytes and an EBV transformed cell line. Each of the three SV callers we used for this dataset, HYDRA, GASV and VariationHunter, returned many results. GASV and HYDRA returned a similar quantity (>13 000 and >12 000, respectively) while VariationHunter returned 843. The callers also returned many results for the whole genome sample. Two callers, GASVPro and VariationHunter, returned >8000 results, and the other two callers, GASV and HYDRA, returned >58 000 and >30 000, respectively.

Without accurate prioritization, a result list with thousands of calls is not particularly useful. Based upon validation of 64 junctions in the target–capture dataset, and 190 deletions in the whole-genome sample, a small fraction of all candidate SVs, the callers did not appear to adequately provide this capability. Caller performance individually (see Section 3.1) and in combination, using agreement between callers, was wanting, especially for the target–capture experiment.

**Fig. 2.**
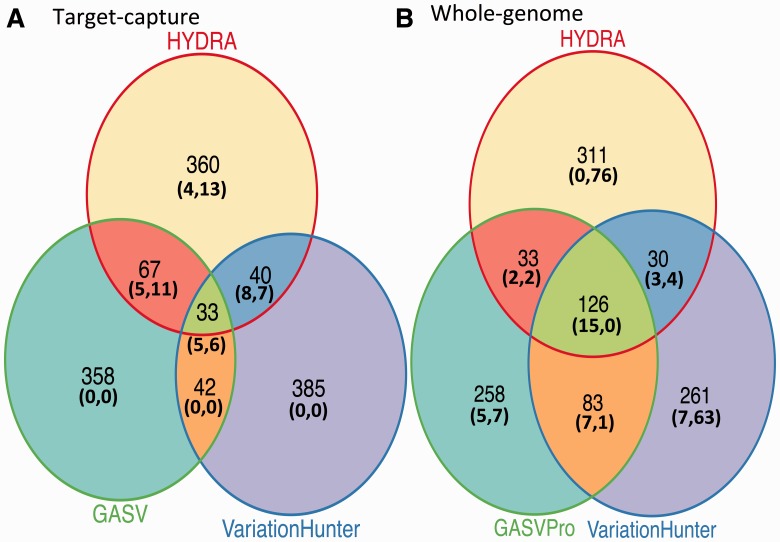
Overlap between the top 500 candidate junctions reported by three methods. GASV is prioritized based upon coverage, GASVPro is prioritized based upon the log likelihood score field, VariationHunter is prioritized based upon the heuristic score field, and HYDRA is prioritized based upon the final weighted support field. Numbers in parentheses represent validated junctions in the top 500, annotated as (true positives, false positives)

In our target–capture dataset, agreement between callers was relatively low and did not serve to distinguish validated true positives from false positives. When considering the top 500 calls from each method, ∼74% were made by only one method, ∼20% were made by exactly two methods and ∼7% were made by all three (Fig. 2A). The existence of calls made by multiple methods raised the possibility of using consensus to circumvent the weaknesses of any individual caller. However, when considering the validated SVs, this prospect did not translate to reality. In the call sets of top 500 candidates, there were nearly equal proportions of validated true positives and false positives called by multiple methods. In these sets, 24 false positives, 63% of those validated and 18 true positives, 69% of those validated, were reported by at least two callers. Additionally, there was no apparent advantage to distinguishing between calls made by exactly two methods and calls made by all three methods. Five out of the 18 true positive calls made by multiple methods (28%) were made by all three methods. Eight out of the 24 false positive calls made by multiple methods (25%) were made by all three methods.

We show results for agreement between HYDRA VariationHunter, and GASVPro for the whole-genome sample because we were able to use GASVPro, a probabilistic version of GASV, in the whole-genome case. When considering the top 500 calls from each method, 55% were made by just one method, 20% were made by exactly two methods and 25% were made by all three (Fig. 2B). Of validated deletions within this set, calls made by multiple methods were enriched for true positives much more than false positives. Twenty true positives, 51% of those validated, were reported by at least two callers, while only seven false positives, 5% of those validated were reported by at least two callers.

Based on these results, using agreement between callers to prioritize candidates was a mixed bag. It was not useful for the target–capture and somewhat useful for the whole-genome results. In the whole-genome sample, calls made by multiple methods were trustworthy compared with calls made by just one method, yet about half of validated positives were not multiple method calls. These data indicated that a different prioritization was needed.

### 3.1 Superior prioritization using likelihood

**Fig. 3.**
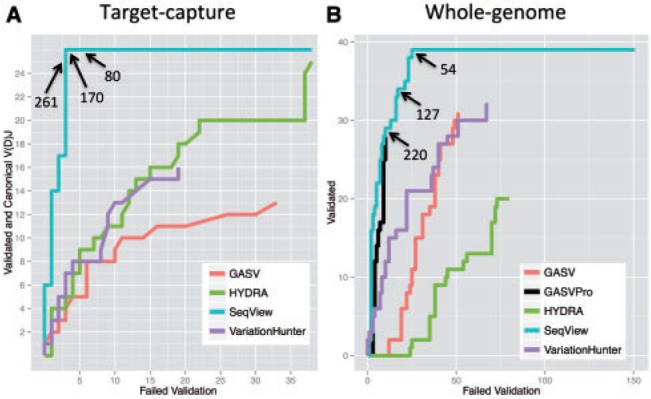
ROC-like plot comparing our method to four existing methods for a subset of validated results. (A) Results for target-capture sequencing. The blue line represents our method, prioritized based upon our likelihood score. Numbers along the blue line indicate our likelihood scores at ranked positions indicated by the arrows (28, 30 and 33). Ranked scores 30–64 all are false positives. Green, red and purple lines represent the other methods tested. GASV is prioritized based upon coverage, VariationHunter is prioritized based upon the heuristic score field and HYDRA is prioritized based upon the final weighted support field. (B) Results for whole-genome sequencing. Colors and candidate prioritizations are the same as panel (A). Since GASVPro works on whole-genome data we show results for this method as well, prioritized by the log likelihood score field. Numbers along the blue line indicate our likelihood scores at ranked positions indicated by the arrows (39, 51 and 64). Ranked scores 65–190 all are false positives

In our target–capture dataset we assigned 26 of the candidate SV junctions originating from the output of HYDRA as true positives and 38 as false positives. This was based upon PCR (52 junctions) or the observation of canonical V(D)J recombination (12 junctions). Most (14) validated junctions were deletions although there were many (9) inversions and a few translocations (3). A table describing the breakdown of all 64 tested junctions is included in the supplement (Supplementary Table S1). We tested the performance of each of the three methods that were compatible with target–capture data against our likelihood score using these 64 junctions (Fig. 3A). The truncated curves for VariationHunter and GASV are owed to our choosing validation junctions based upon the candidate list from HYDRA. As a result, some of these SVs, both true positives and false positives, did not appear in the results from the other two methods. The ROC-like curve shows the superiority of our scoring method, with the three other methods performing similarly to each other by comparison. The plot is ROC-like rather than a true ROC because it only contains validated junctions, not all of the many thousands of results returned by the tested methods. That said, likelihood scores of the top 500 candidates from each method suggested that our validation set included most of the positive events present in the target–capture dataset (Supplementary Figure S2). Of note, the three especially high scoring false positives in Figure 3A, signified by horizontal segments at *y*-axis positions 6, 14 and 17, may, in fact, be PCR failures. All three occurred in the same sample (pre-B ALL) and PCR produced multiple bands in both control and sample DNA. They are assigned as false positives because we could not decisively conclude that they were real based upon the validation.

To assess performance on the whole-genome sample, we restricted results of HYDRA, GASV, GASVPro and VariationHunter to high confidence deletions >8-kb long. We used this cutoff because the majority, 81%, of the validation regions were >8-kb long. We focused on the top 100 calls meeting these criteria from HYDRA and VariationHunter, and all such calls from GASV (82 total) and GASVPro (39 total). As with the target–capture data, we compared the prioritization of validated positive and negative deletions from existing methods to our likelihood score (Fig. 3B). For the whole-genome analysis we were also able to incorporate GASVPro results, as this tool is compatible with whole-genome data. From this dataset we assigned 39 of the candidate deletions originating from the output of one of the four tools as true positives and 151 as false positives. Only deletion candidates >8-kb long were considered for follow-up due to our validation technique, which was based on read–depth. Again, the ROC-like curve demonstrated the advantage of our method. Notably, GASVPro performs quite well in relation to the other three existing methods, and only slightly underperforms relative to our likelihood score. Based upon this result we recommend our tool most avidly for reprioritizing candidate deletions from HYDRA, GASV and VariationHunter in whole genome data. We recommend a cut-off score of 100, a compromise between our whole-genome and our target–capture results. A cutoff score of 170 is optimal for our target–capture experiment, as this is the score at the inflection point in Figure 3A, however it is too conservative for our whole-genome results, which scored somewhat lower in general than the target–capture results (Supplementary Figure S3). A score of 100 enables us to identify 34 of 39 (87%) true positives while still ruling out 134 of 151 false positives (89%) in the whole-genome data (Fig. 3B).

### 3.2 Why the likelihood approach works

**Fig. 4.**
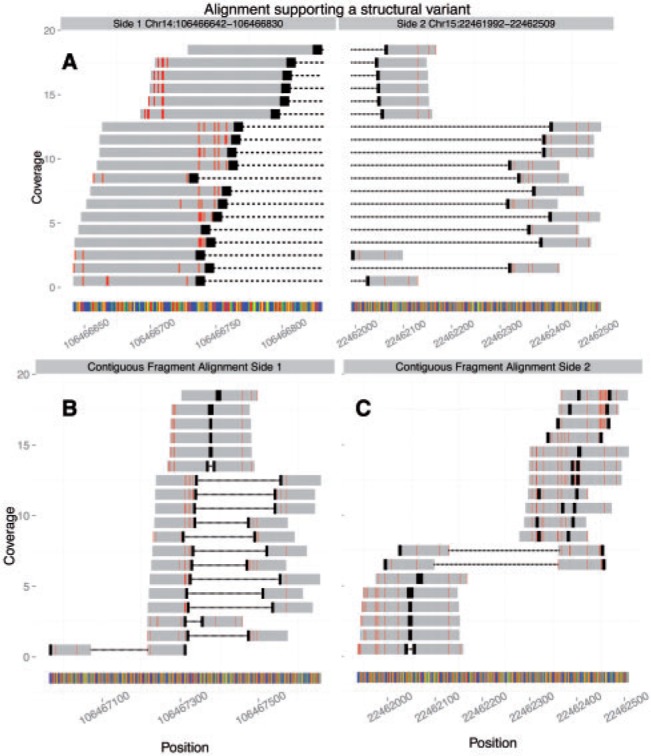
Visualizing a false positive. (A) Realignment of read–pairs from a PCR-validated false positive to a repetitive region of chromosome 15 and the IGH locus on chromosome 14. Each row shows one read–pair, with 19 junction spanning read–pairs shown in total. Red indicates mismatches. Black caps signify the 3′-end of the read. For pairs with overlapping read alignments the black cap is indicative of the 3′-end of the forward read. Read–pairs are connected with lines (with a short gap at the junction). (B) Same reads as (A) aligned to an expanded reference section from the left side of (A). (C) Same reads as (A) aligned to an expanded reference section from the right side of (A)

The likelihood approach worked because it focused on one simple characteristic that defined events that failed to validate: realignment of supporting reads was consistent with the unrearranged reference (Fig. 4). For instance, the event shown in Figure 4 is a false positive chromosomal translocation. It was called in our Ramos cell-line by GASV and HYDRA. Figure 4A depicts the reads supporting the candidate deletion as demonstrated by the majority of the read–pairs mapping one end to each of the two loci and oriented towards each other. Figure 4B and C show realignments of these same reads in configurations consistent with an unrearranged sequence. Both ends of read–pairs map well to side 1 (Fig. 4B) of the alleged junction and to a lesser, but still noticeable extent, side 2 (Fig. 4C), demonstrating that these two loci are homologous. Without the information in Figure 4B and C, it would be difficult to know that this candidate SV is a false positive. It is through our realignment scheme that we obtain the information to do this comparison, information that upstream SV callers cannot access since they do not manipulate read alignments, and instead rely on an alignment file ‘as is’. The score provides a one number summary useful for prioritization.

### 3.3 Importance of visualization

Visualization is not strictly necessary to use our method but there are reasons it is a feature. For one, the research community commonly integrates visualization of read alignments into next-generation sequencing data-analysis pipelines. Second, available viewers are not capable of also realigning reads, so they will not be able to show our realignments, which are crucial to conveying the information in our score. Since we are unaware of a viewer that is also an aligner our provided visualization is likely the most convenient way to produce alignment views generated by our method. Viewing also provides more detail than a univariate score, including coverage, error locations within reads and read orientations.

**Fig. 5.**
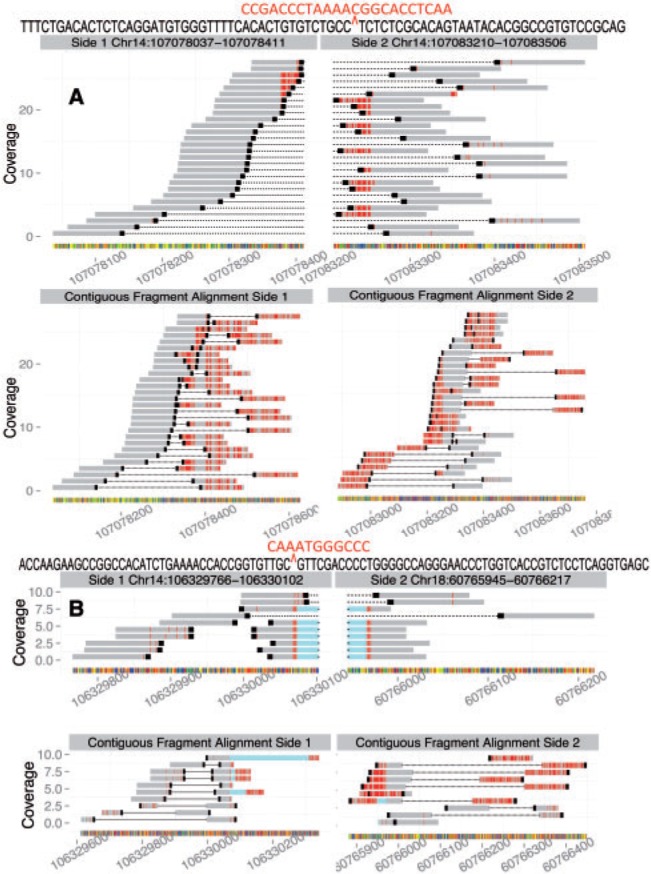
Observing added bases at the breakpoints of two events. (A) Top: realignment of reads from the ARH-77 cell line to reference positions indicating an interstitial deletion with inserted bases on chromosome 14. Black caps indicate the 3′-end of the reads. The extra bases may be seen as red at the ends of the reads abutting the breakpoint, as these are mismatches between the reference and the reads. Read–pairs are connected with lines (with a short gap at the junction). The sequence of a split-read is shown above the alignment, with added bases in red above bases matching the two sides of the reference. Bottom: the same read–pairs as in panel (A), aligned to expanded sections of the reference sequences from the two sides of the above junction. (B) Top: realignment of reads from the DB cell line indicating a *t*(14;18) chromosomal translocation. Rows with light-blue bridging the two sides of the junction are split-reads. The added bases may be seen as red at the flanks of split positions. The sequence of a split-read is shown above the alignment, with added bases in red above bases matching the two sides of the reference. Bottom: same reads as (B) top aligned to expanded sections of the reference from the two sides of the above junction

To illustrate the point, consider two validated positive junctions, an interstitial deletion in the ARH-77 cell line (Fig. 5A) and a *t*(14;18) junction on der(14) in the DB cell line (Fig. 5B). In both cases we observed that the nucleotides right at the breakpoint did not match the reference sequence. In Figure 5A, mismatching breakpoint sequence is identifiable as stretches of red at the ends of most aligning read–pairs. In Figure 5B, mismatching breakpoint sequence is identifiable within split-reads crossing the deletion junction. Flanking the split, illustrated with light blue bars, are thin areas of red, representing mismatches. These mismatching bases are a normal phenomenon resulting from V(D)J recombination and are owed to the activity of the enzyme terminal deoxynucleotidyl transferase (TdT). Above the alignments we include the sequence of a split-read crossing each junction, with bases matching the reference sequence at the two involved loci in black and mismatched, untemplated bases in red. We include Sanger Sequencing results for these two junctions in the Supplementary Material (section ‘Sanger Sequencing’). TdT is known to add untemplated bases at the junctions of juxtaposed V(D)J coding segments in B and T-cell lymphocytes. Visualizing these junctions facilitated the identification of TdT activity, and though it was not necessary to confirm the events, added to understanding the events at a molecular level.

## 4 DISCUSSION

We have shown that our method for distinguishing false positives from true SVs is a useful addition to upstream analysis tools. It enables assessment of candidate SVs, either individually, as part of manual inspection of alignments (Figs 4 and 5), or in summary with a probability-based score. Without our post-processing visualization and scoring, we would have had an unmanageably large list of candidate SVs replete with artifacts. Although our method relies on assumptions known not to hold exactly, approximate model permits the implementation of a fast and stable algorithm that greatly improves downstream results. Extending our method to avoid depending on these assumptions is a matter of future work.

Our method retains the sensitivity to detect events within repeat elements demonstrated on segmental duplications. Our method is an improvement over existing tools for distinguishing likely artifacts within these regions (Fig. 3). Of note in the ROC-like plot depicting our target–capture data (Fig. 3A), the slightly decreased slopes of GASV and VariationHunter in comparison to HYDRA are likely not due to true underperformance but rather to selection bias in our choice of candidates to validate. The important feature of the plot is to demonstrate our superior classification of candidates in the repetitive loci present in our data. Accordingly, our scheme successfully identified 19 SVs having at least one junction within a segmental duplication (13 in the target-capture and six in the whole-genome sample) while ruling out another 102 (29 in the target capture and 73 in the whole-genome sample) candidate junctions within a segmental duplication that did not validate (Supplementary Material). Because our method serves not only to rule out candidates in repetitive DNA, but also to identify true events in these regions, it is not simply a proxy for sequence masking algorithms like repeatMasker (Smit *et al.*, 1996–2010).

Visual inspection of read alignments is an important means for candidate SV follow-up for good reason. Resolving sequences from short reads generated from repetitive regions of the genome is challenging. As such, skepticism towards the initial results of an analysis pipeline designed to use these alignments to detect SVs is warranted and a post-candidate-list-analysis step appears to be the de-facto norm. Alignment viewers, such as the Integrative Genome Viewer (IGV) (Robinson *et al.*, 2011), are popular, but not specifically designed for the task. In fact, there is a general lack of systematic approaches for candidate SV analysis. Furthermore, statistical models of the SV detection problem fail to incorporate important information, such as cycle-dependent error rates. Our method models this but none of the others we tested do so.

We provide our visualization scheme in recognition of the large role alignment viewers have come to play. Inspecting the three alignments per candidate SV necessary to understand our likelihood score seems likely to be a part of any pipeline that would integrate our method. As such, this feature is convenient since it will be easier to generate visualizations using our tool rather than to do so independently, a process that would involve both realignment and side-by-side viewing of realignments in a viewer. Our visualization also provides more information than is possible with a one-number score. This is apparent by the fact that visualization helped to identify nucleotides added by the enzyme terminal deoxynucleotidyl transferase (TdT) at the junctions of juxtaposed V(D)J coding segments in our target–capture experiment (Fig. 5). Of note, for those uninterested in visualization and only seeking a quick result, we also provide a faster implementation of our score that skips plotting and only performs a fraction of the full realignment (described further in the Supplementary Material section ‘R Package Notes: Implementation of the probability score’).

High-throughput short-read technology has facilitated single-base inspection of much of the human genome. However, we believe that manual scrutiny of repetitive region alignments will remain popular and necessary until the ambiguity surrounding these alignments is resolved with superior technology. This likely will remain especially true in cases where the standard reference genome is known to be an untrustworthy representation of the genome in question, as is the case with cancer genomes and cell lines. Here we have handled these types of genomes and shown that likelihood scoring and visual inspection of candidate SVs is an effective means to eliminate a common type of artifact appearing in lists of events generated by upstream analysis techniques.

## Supplementary Material

Supplementary Data
