# Supplementary material for: Visualization and probability-based scoring of structural variants within repetitive sequences
Source: Bioinformatics. 2014 Feb 4;30(11):1514–21. doi: 10.1093/bioinformatics/btu054 (PMC4029030; doi:10.1093/bioinformatics/btu054)
Supplement: Supplementary Data [file supp_30_11_1514__index.html]

Visualization And Probability-Based Scoring of Structural Variants within Repetitive Sequences — Visualization and probability-based scoring of structural variants within repetitive sequences — Visualization and probability-based scoring of structural variants within repetitive sequences — Supplementary Data 

# Visualization and probability-based scoring of structural variants within repetitive sequences

## Supplementary Data

files

**Files in this Data Supplement:**

- Supplementary Data - docx file
- Supplementary Data - xlsx file
