# Supplementary material for: Visualization and probability-based scoring of structural variants within repetitive sequences
Source: Bioinformatics. 2014 Feb 4;30(11):1514–21. doi: 10.1093/bioinformatics/btu054 (PMC4029030; doi:10.1093/bioinformatics/btu054)
Supplement: Supplementary Data [file supp_btu054_1106onlinemethods.docx]

Supplemental Methods

## Experimental approach: Target-capture

Our experimental approach included a pull-down assay and subsequent paired-end Illumina sequencing. For our pull-down assay, we used biotinylated RNA baits targeting 440 genomic loci immediately adjacent to coding V(D)J segments, designed for five-fold coverage of Agilent SureSelect bait tiling (Agilent Technologies, Santa Clara CA). To this end, 2461 120mer baits were designed to cover 200 bp windows, fully including the recombination signal sequences (RSS), using eArray. We performed the pull-down on 11 samples; six primary tumors; four cancer cell lines (American Type Culture Collection, Manassas, VA); and one EBV-transformed lymphoblastoid line (HuRef, Coriell Institute, Camden, NJ). We obtained gDNA from viable cells or fresh frozen primary cells by phenol-chloroform extraction and ethanol precipitation. HudsonAlpha Institute for Biotechnology (Huntsville, AL) sequenced our indexed library in one lane of an Illumina HiSeq generating 7,339,278 100 bp paired-end reads of median insert size 254 and median coverage 173X across the 440 bait regions. A full description of IRB approval, samples, probe design, library prep using the pull-down assay, and sequencing is available in another manuscript (Halper-Stromberg, 2013).

## Experimental approach: Whole-genome

We sequenced a single lymphoblast cell-line using a paired-end protocol in one lane of an Illumina HiSeq, generating 473,071,320 101 bp reads of median insert size 270 and an average genome-wide coverage of ~15x.

## Generating candidate lists

Preparing the input files for each of the existing tools varied by tool. For HYDRA this involved removing from our alignment file read-pairs with an orientation and insert size that would qualify as expected, i.e. partners facing each other and separated by no more than 800 bases of intervening sequence. We used two different short read aligners for this step, in an attempt to be thorough in the filtering of normal read-pairs. First we aligned reads to hg19 using Bowtie, ridding our resultant bam files of expectedly aligned pairs. We subsequently aligned remaining reads using Burrows-Wheeler Aligner (BWA), again ridding the resultant bam file of expectedly aligned reads. We merged alignments reported in the XA tag of this output with the primary alignments in our bam file using a python script (available with our R package on github).

We were unable to use the GASVPro algorithm on our target-capture sequences, as it makes assumptions about read depth over non-rearranged loci that are only applicable to whole genome sequencing experiments. As a result, we instead generated a candidate SV list using GASV, an intermediate tool in the GASVPro pipeline. The GASV software accepts unfiltered bam files, requiring only that two separate bams be provided, one with unique alignments and one with ambiguous multiple alignments. We thus aligned reads using BWA and used our python script to generate the two required bam files.

For VariationHunter, preprocessing involved mapping reads to the hg19 reference using the MrFast aligner. The output from this aligner only is compatible with VariationHunter and is the reason we did not use BWA.

## Validation: Target-capture

We filtered the candidate list from HYDRA down to 70 junctions using three filter criteria, requiring that junctions: 1) be supported by at least 5 read-pairs, 2) overlap with at least one of our bait sequences and 3) be supported by read-pairs aligning with relatively few mismatches (supporting read-pairs were required to have a mean edit distance < 2.6). Of these 70, we discarded 6 junctions supporting canonical V(D)J (these junctions were still assigned true positive status and were included in the ROC, Fig 2 of the main paper). Of the remaining 64, 7 supported a chromosomal recombination between chr14 and chr16. We noted upon manual inspection that the two loci were homologous and we discarded all but one of these candidates to test. Of the remaining 58, we noticed that 10 of the junctions could be collapsed down to 5 based upon overlap between called junctions in the same sample. It is possible that each pair of overlapping junctions included two independent, yet nearly identical events, but we were not prepared to test such a hypothesis. It should be noted also that we kept 4 pairs of junctions that were close to each other in the same sample because in these cases differently oriented read-pairs supported both an inversion and a deletion. Of those remaining, we discarded 1 event that seemed likely to be a PCR artifact based on the fact that it was supported by 5 read-pairs and both ends aligned at the same start positions, respectively. Next we discarded 7 events where the 5’ and 3’ junctions were homologous. Since we performed this step before developing our visualization and scoring technique, we went about it unsystematically: We used the Blat tool available from the UCSC genome browser to query sequences taken from read alignment locations within candidate event loci. If Blat returned hits at the two loci of a called event we discarded the event. Our final set consisted of the remaining 45 junctions plus 7 junctions not meeting our initial 3 criteria. The junctions added back to the list included: 1 interstitial deletion event overlapping a called inversion in the pre-T ALL sample which we noticed on chr14:106,351,900 while inspecting the inversion event, 3 events indicating chromosomal recombination between chr14 and chr15, and 2 events indicating interstitial deletions on chr14. The last 5 mentioned were all of biological interest, and although they did not meet the edit distance filter they passed the other two of our initial filters. Lastly, we added back the der(18) junction in the DB sample. This junction did not meet the read-depth filter but we strongly suspected it was real based on the evidence supporting the t(14;180) der(14) junction. For PCR validated junctions, we assigned a candidate as positive if we were able to amplify a fragment of DNA crossing the SV junction and no similar fragment in control DNA. For canonical V(D)J validated junctions, we assigned a candidate as positive if its junction indicated the juxtaposition of two V(D)J coding segments or two recombination signal sequence (RSS) sites in a manner consistent with normal immunoglobulin or T-cell receptor construction.

## Validation: Whole-genome

We filtered the four candidate lists generated by HYDRA, VariationHunter, GASV, and GASVPro using two filter criteria, requiring that junctions 1) indicate an interstitial deletion and 2) involve a stretch of sequence at least 8 Kb in length. From these filtered lists we retained the top 100 candidates from HYDRA and VariationHunter and all of the remaining GASV and GASVPro candidates, 82 and 39 respectively. These candidates yielded 190 unique deletion regions once we accounted for overlap between lists. Read-depth CNV calling was performed on the paired-end sample as well as a pair of technical-replicates generated from the same aliquot and run using a single-end protocol. The single-end technical replicates were run on the same flow cell at the same time, in adjacent lanes of an Illumina HiSeq, generating 159,027,554 and 140,918,922 101 bp reads, respectively. Read-depth calls were generated using ERDs and CNVnator for the paired-end sample, and CNVnator for the two single-end samples. A candidate was assigned as positive based upon two criteria: 1) Both loci involved in the candidate deletion junction were within the region of a deletion call made by either CNVnator or ERDs in the paired-end sample, and the candidate overlapped at least 50% of the called region. 2) The candidate overlapped a deletion call made by CNVnator in at least one of the two single-end samples.

## Smith-Waterman alignment parameters

We connected the Smith-Waterman algorithm parameters with our probability scoring by using position-specific substitution rates (as implemented in the Biostrings function we use to perform the alignment). The position-specific substitution rates in the Smith-Waterman alignment match the position-specific error probabilities in our binomial model. Alignment probabilities for each base may be expressed as log-likelihood scores. Our Smith-Waterman match scores are therefore log(p_match_/p_observed alignment occurring randomly_) and our mismatch scores are log(p_mismatch_/p_observed alignment occurring randomly_). A more detailed description is provided in the paper upon which the position-specific Smith-Waterman algorithm is based*([Malde, 2008](#_ENREF_1)). In practice, this translated to match scores hovering around 2 and mismatch scores ranging from about -4 to -8. In addition, we connected the Smith-Waterman gap-open penalty to our indel rates by making this penalty log(mean(position-specific indel rates)), which translates to a gap open penalty around -10 using our data.

*Malde, K. (2008) The effect of sequence quality on sequence alignment, *Bioinformatics*, 24, 897-900.

## R Package Notes: Implementations of the probability score

The vignette for our package is located in the rendered markdown file displayed at <https://github.com/Eitan177/targetSeqView> and in the pdf file located in the vignettes folder. We provide two implementations of our scoring procedure. The first implementation, accessed using the function ‘fullScoreAndView’, performs Smith-Waterman realignment of reads in the three configurations described in the main text. The probability score is constructed as detailed in section 2.4 of the main text and realigned reads are formatted for visual inspection, such as those shown in Figures 4 and 5 in the main text. We provide a 2^nd^ experimental implementation of our score, accessed using the function ‘quickScore’, for faster results. There are three differences between our ‘quickScore’ scheme and the one described in the main text:

1) We realign only one read per read-pair to each of the two unaffected-references, the one for which alignment at the reference in question supports a contiguous-fragment-supporting scenario.

2) We perform no SV-supporting realignment, but instead trust the alignment information in the alignment file, based on the MD flags and the cigar strings, to generate probability scores.

3) Each read-pair is allowed to support only one contiguous fragment. This is decided based upon which contiguous-fragment-supporting alignment generates the best Smith-Waterman alignment score.

The ‘quickScore’ scheme is faster (performs 1/3 the number of realignments and does not do formatting for plotting) and may prove a more practical option for evaluating a large SV list with 1000s of candidates. If we take the example of the read-pair shown in Fig 1 the ‘quickScore’ scheme would extract mismatch/indel information in part A from the bam file and perform realignment only on the green read in part B, and the blue read in part C where as our main score would perform realignment on all reads shown in Fig 1. The rationale for not realigning all reads is that the results of Smith-Waterman and the results of short-read aligners are likely nearly the same in cases where reads match the reference well enough such that a short-read aligner was able to map them.

## Sanger Sequencing

PCR results for the event depicted in Fig 4A (nucleotides matching the reverse-complement of the split-read are in blue):

NNNNNNNNNNNNNNNATGCNNNNGCCGCCNTGGCGGCCGCGGGAATTCGATCCCTCCCTCAAGAGTCGAGTCACCATATCAGTAGACACGTCCAAGAACCAGTTCTCCCTGAAGCTGAGCTCTGTGACCGCTGCGGACACGGCCGTGTATTACTGTGCGAGAGATTGAGGTGCCGTTTTAGGGTCGGGGCAGACACAGTGTGAAAACCCATATCCTGAGAGTGTCAGAAACGCCAGGAAGGAGGCAGCTGTACTGGCATGGAATCACTAGTGAATTCGCGGCCGCCTGCAGGTCGACCATATGGGAGAGCTCCCAACGCGTTGGATGCATAGCTTGAGTATTCTATAGTGTCACCTAAATAGCTTGGCGTAATCATGGTCATAGCTGTTTCCTGTGTGAAATTGTTATCCGCTCACAATTCCACACAACATACGAGCCGGAAGCATAAAGTGTAAAGCCTGGGGTGCCTAATGAGTGAGCTAACTCACATTAATTGCGTTGCGCTCACTGCCCGCTTTCCAGTCGGGAAACCTGTCGTGCCAGCTGCATTAATGAATCGGCCAACGCGCGGGGAGAGGCGGTTTGCGTATTGGGCGCTCTTCCGCTTCCTCGCTCACTGACTCGCTGCGCTCGGTCGTTCGGCTGCGGCGAGCGGTATCAGCTCACTCAAAGGCGGTAATACGGTTATCCACAGAATCAGGGGATAACGCAGGAAAGAACATGTGAGCAAAAGGCCAGCAAAAGGCCAGGAACCGTAAAAAGGCCGCGTTGCTGGCGTTTTTCCATAGGCTCCGCCCCCCTGACGAGCATCACAAAAATCGACGCTCAAGTCAGANGTGGCGAAACCCGACAGGACTATAAAGATACCAGGCGTTTCCCCCTGGAAGCTCCCTCGTGCGCTCTCCTGTTCCGACCCTGCCGCTTACCGGATACCTGTCCGCCTTTCTCCCTTCGGGAAGCGTGGCGCTTTCTCATAGCTCACGCTGTNNNATCTCAGTTCGGTGTNNNCGTTCGCTCCAAGCTGGGCTGTGTGCACGACCCCCGTTCAGCCCGACGCTGCGCNATCNGTACTATCGTCNTGAGTCCANCCGGTANACNCGACTTATCGCCNCTGGNANCAGCCACTGGNAACNNNTTANNNNNNNNAN

PCR result for the event depicted in Fig 4B (nucleotides matching the split-read are in blue):

NNNNNNNNNNGNNNNNGCCGCCNTGGCGGCCGCGGGAATTCGATTACTCAATGCCCACACCTGATGTGCTAATCACTGGGTGAATTATTTTCACTTCTGTGAACAAGGCCTCTGCTATCGCTTCACCAAGAAGCCGGCCACATCTGAAAACCACCGGTGTTGCCAAATGGGCCCGTTCGACCCCTGGGGCCAGGGAACCCTGGTCACCGTCTCCTCAGGTGAGTCCTCACCACCCCCTCTCTGAGTCCACTTAGGGAGACTCAGCTTGCCAGGGTCTCAGGGTCAGAGTCTTGGAGGCATTTTGGAGGTCAGGAAAGAAAGCCGGGGAGAGGGACCCTTCGAATGGGAACCCAGCCTGTCCTCAATCACTAGTGAATTCGCGGCCGCCTGCAGGTCGACCATATGGGAGAGCTCCCAACGCGTTGGATGCATAGCTTGAGTATTCTATAGTGTCACCTAAATAGCTTGGCGTAATCATGGTCATAGCTGTTTCCTGTGTGAAATTGTTATCCGCTCACAATTCCACACAACATACGAGCCGGAAGCATAAAGTGTAAAGCCTGGGGTGCCTAATGAGTGAGCTAACTCACATTAATTGCGTTGCGCTCACTGCCCGCTTTCCAGTCGGGAAACCTGTCGTGCCAGCTGCATTAATGAATCGGCCAACGCGCGGGGAGAGGCGGTTTGCGTATTGGGCGCTCTTCCGCTTCCTCGCTCACTGACTCGCTGCGCTCGGTCGTTCGGCTGCGGCGAGCGGTATCAGCTCACTCAAAGGCGGTAATACGGTTATCCACAGAATCAGGGGATAACGCAGGAAAGAACATGTGAGCAAAAGGCCAGCAAAAGGCCAGGAACCGTAAAAAGGCCGCGTTGCTGGCGTTTTTCCATAGGCTCCGCCCCCCTGACGAGCATCACAAAAATCGACGCTCAAGTCAGANGTGGCGAAACCCGACAGGACTATAAAGATACCAGGCGTTTCCCCCTGGAAGCTCCCTCGTGCGCTCTCCTGTTCCNACCCTGCCGCTTACC

## Table S1: Breakdown of target-capture validation set by SV class

|  | Deletions | Inversions | Translocations | Insertions |
| --- | --- | --- | --- | --- |
| Validated | 14 | 9 | 3 | 0 |
| Failed Validation | 9 | 6 | 8 | 15 |
| Validation Rate | 0.609 | 0.6 | 0.273 | 0 |

## Supplemental Figures

**Figure S1: ROC-like plot comparing our method with and without making accommodation for apparent Snps.** A) Results for target-capture sequencing. The dotted blue line represents our method as presented in the main text and the dotted magenta line represents our method after accounting for apparent Snps as defined by concordant mismatches at the same position across multiple reads. All other lines and information are the same as shown in Figure 3A of the main text. Our method for taking Snps into account involved assigning each instance of mismatches concordant across at least two reads to a separate category than other mismatches. The number of instances of these observations per alignment configuration, interpreted as the number of Snps observed, were assigned a probability equal to: *p_s_^is^* where *p_s_* was the probability of observing concordance and *i_s_* was the observed number of concordances. We estimated *p_s_* based upon the frequency of concordance in normal alignments in each experiment (0.0053 for the target-capture and 0.0036 for the whole-genome experiment). This new ‘Snp’ probability, one per configuration, was then multiplied by the probability calculated according to our method defined in the main text to come up with a new probability for each configuration. As demonstrated by the Figure, this new probability had little effect on performance. B) Result for whole-genome sequencing. The blue line represents our method as presented in the main text and the magenta line represents our method after accounting for apparent Snps as described in part A. All other lines and information are the same as shown in Figure 3B.

## Supplemental Figures Continued

**Figure S2: Likelihood scoring of untested candidates and ranking of tested candidates** A) Distributions for untested candidate events in the top 500 call sets for the target-capture experiment B) Rank of tested candidates within top 500 candidates from HYDRA, GASV, and VariationHunter target-capture result lists

**Figure S3: Distributions for all tested candidate events in the target-capture and whole-genome experiments, separated by validation status.**
